# Editorial: Food-based dietary guidelines

**DOI:** 10.3389/fnut.2025.1724966

**Published:** 2025-10-31

**Authors:** Carla Gonçalves, Grigoris Risvas, Norman Temple

**Affiliations:** ^1^Departamento de Biologia e Ambiente, Universidade de Trás-os-Montes e Alto Douro, Vila Real, Portugal; ^2^Centro de Investigacao e de Tecnologias Agro-Ambientais e Biologicas (CITAB), Universidade de Trás-os-Montes e Alto Douro, Vila Real, Portugal; ^3^Instituto de Saude Publica (ISPUP), Universidade do Porto, Porto, Portugal; ^4^Centro de Investigacao em Tecnologias e Servicos de Saude (CINTESIS), Porto, Portugal; ^5^Aegean College, Athens, Greece; ^6^Athabasca University, Athabasca, AB, Canada

**Keywords:** food based dietary guidelines, food guide, food literacy, healthier diet, public health

Food-based dietary guidelines (FBDGs) are among the most widely used public health tools for translating nutrition science into practical advice for populations. By offering culturally adapted and evidence-based recommendations on food choices, portion sizes, and dietary patterns, FBDGs aim to promote health, prevent disease, and increasingly, address the sustainability of food systems. However, despite their global spread (more than 100 countries now have national FBDGs), challenges remain in ensuring their accessibility, cultural adaptability, methodological rigor, and integration into coherent food and nutrition policies.

This Research Topic, *Food-based dietary guidelines*, brings together diverse contributions that address key aspects of the design, application, and evaluation of dietary guidelines across different populations and settings. Together, the articles collected here highlight both persistent gaps and innovative approaches in the development and implementation of FBDGs, offering lessons that are highly relevant for researchers, policymakers, and practitioners.

The affordability of healthy eating is a cornerstone for the successful implementation of dietary recommendations, particularly in low- and middle-income countries. In their study, Gonzabay-Parrales et al. illustrated the stark economic barriers faced by families in Quito and Guayaquil, in Ecuador. The authors demonstrated that following a healthy diet requires almost half of the basic monthly salary, making it inaccessible to many households. This work underscores the urgent need for policies that improve access to healthy foods and incentivize local trade between producers and consumers. Without addressing economic constraints, FBDGs risk remaining aspirational rather than actionable.

Several contributions have highlighted how actual dietary behaviors align—or fail to align—with recommended guidelines. In Sweden, the study by Mulkerrins et al. assessed food intake among young adults with different dietary practices. Despite differences in consumption patterns—such as higher intakes of legumes and plant-based substitutes among vegans and vegetarians—the overall adherence to FBDGs was low, particularly for fruits, vegetables, nuts, and whole grains. Similarly, the study by Rohm et al., based on the third Bavarian Food Consumption Survey, revealed that a large proportion of adults in Bavaria, Germany, do not meet FBDG recommendations, echoing findings from two decades earlier. Although some improvements were observed, such as reduced meat and soft drink consumption, deficiencies in fruit, vegetable, and whole grain intake persist, with potential risks of nutrient insufficiency. Together, these studies remind us that dietary guidelines are only as effective as the population's capacity and willingness to follow them. Monitoring dietary behaviors over time remains essential for evaluating the impact of guidelines and identifying priority areas for intervention.

Another group of articles focused on tools to assess diet quality and their alignment with FBDGs. In Canada, Panahimoghadam et al. compared the Healthy Eating Index-Canada, the Diet Quality Index-International, and the Healthy Eating Food Index 2019. The authors showed that these indices vary in their discriminatory power and agreement, leading to different interpretations of children's diets. Their call for consensus highlights the importance of methodological alignment to ensure coherent dietary monitoring and policy guidance. In this sense, in the United States, Katz et al. introduced an adaptation of the Healthy Eating Index to better reflect multicultural dietary patterns. This innovation allows recognition of nutritional quality across diverse diets, moving toward more inclusive and equitable assessment tools.

Portion size guidance is a core yet often underexplored element of FBDGs. Two studies in this Research Topic addressed this issue directly. The article from Salesse et al. examined the methodological approaches to deriving portion sizes across 96 countries, finding limited variation by method but some regional differences, particularly for fish and shellfish. In a second, complementary study, the same authors revealed substantial inconsistencies across regions, especially in definitions and classifications of food groups. Both studies highlight the potential of harmonizing portion size recommendations to improve clarity and comparability. Supporting these efforts, Fallata et al. provided a structured approach for creating reliable food atlases. These visual tools, which include culturally relevant portion sizes and utensils, can play an important role in dietary assessment and in communicating guidelines to diverse populations once validated through further study.

Finally, two articles extended the discussion to the policy arena. The article from De Matteu Monteiro et al. highlighted the potential of risk–benefit assessment as a structured, evidence-based approach to guiding food and nutrition policy. Despite methodological advances, the translation of such assessments into concrete policies remains limited, calling for stronger integration between science and regulation. In Southeast Asia, Thanh Nguyen et al. evaluated national strategies against international standards. While progress has been made, important gaps remain, particularly in the inclusion of interventions for women and adolescents. This work emphasizes the importance of aligning national policies with global evidence while maintaining sensitivity to local contexts to ensure progress on reducing non-communicable diseases.

The contributions to this Research Topic underscore the complexity of designing, implementing, and evaluating FBDGs in a rapidly changing food environment. They demonstrate that, while methodological progress is being made—through harmonized portion size recommendations, improved diet quality indices, and adaptive tools—significant challenges remain in terms of affordability, cultural inclusivity, and policy alignment.

While the articles included in this Research Topic provide valuable insights into diet affordability, adherence, methodological frameworks, and policy alignment, other important aspects raised in the original call remain underexplored. Future research should address how best to communicate dietary recommendations through effective visual designs, the role and placement of ultra-processed foods within FBDGs, and additionally, the integration of traditional dietary patterns, social food behaviors, and culturally embedded practices. This integration is key to ensuring the relevance and uptake of guidelines across diverse populations. Finally, the incorporation of environmentally sustainable advice into FBDGs—balancing health, culture, and planetary boundaries—should be prioritized to align dietary recommendations with the urgent goal of transforming the food system. By advancing these areas, future research, with greater interdisciplinary collaboration, harmonization of methods across regions, and stronger integration with policies, can help ensure that FBDGs remain dynamic, inclusive, and impactful tools for improving public health, equity, and environmental sustainability.

